# Enzyme replacement therapy: efficacy and limitations

**DOI:** 10.1186/s13052-018-0562-1

**Published:** 2018-11-16

**Authors:** Daniela Concolino, Federica Deodato, Rossella Parini

**Affiliations:** 1 Department of Medical and Surgical Science, Pediatric Unit, University “Magna Graecia”, Catanzaro, Italy; 2 Division of Metabolic Disease, Bambino Gesù Children’s Hospital, IRCCS, Rome, Italy; 3 UOS Malattie Metaboliche Rare, Clinica Pediatrica dell’Università Milano Bicocca, Fondazione MBBM, ATS Monza e Brianza, Via Pergolesi 33, 20900 Monza, Italy; 4 San Raffaele Telethon Institute for Gene Therapy (SR-TIGET), IRCCS San Raffaele Scientific Institute, Milan, Italy

**Keywords:** Enzyme replacement therapy, ERT, Mucopolysaccharidosis, MPS

## Abstract

Enzyme replacement therapy (ERT) is available for mucopolysaccharidosis (MPS) I, MPS II, MPS VI, and MPS IVA. The efficacy of ERT has been evaluated in clinical trials and in many post-marketing studies with a long-term follow-up for MPS I, MPS II, and MPS VI. While ERT is effective in reducing urinary glycosaminoglycans (GAGs) and liver and spleen volume, cartilaginous organs such as the trachea and bronchi, bones and eyes are poorly impacted by ERT probably due to limited penetration in the specific tissue. ERT in the present formulations also does not cross the blood–brain barrier, with the consequence that the central nervous system is not cured by ERT. This is particularly important for severe forms of MPS I and MPS II characterized by cognitive decline. For severe MPS I patients (Hurler), early haematopoietic stem cell transplantation is the gold standard, while still controversial is the role of stem cell transplantation in MPS II. The use of ERT in patients with severe cognitive decline is the subject of debate; the current position of the scientific community is that ERT must be started in all patients who do not have a more effective treatment. Neonatal screening is widely suggested for treatable MPS, and many pilot studies are ongoing. The rationale is that early, possibly pre-symptomatic treatment can improve prognosis. All patients develop anti-ERT antibodies but only a few have drug-related adverse reactions. It has not yet been definitely clarified if high-titre antibodies may, at least in some cases, reduce the efficacy of ERT.

## Background

Enzyme replacement therapy (ERT), based on the periodic intravenous administration of specific enzymes produced with recombinant DNA technology, is at present the most appropriate available therapy for several lysosomal storage disorders.

**Fig. 1:**
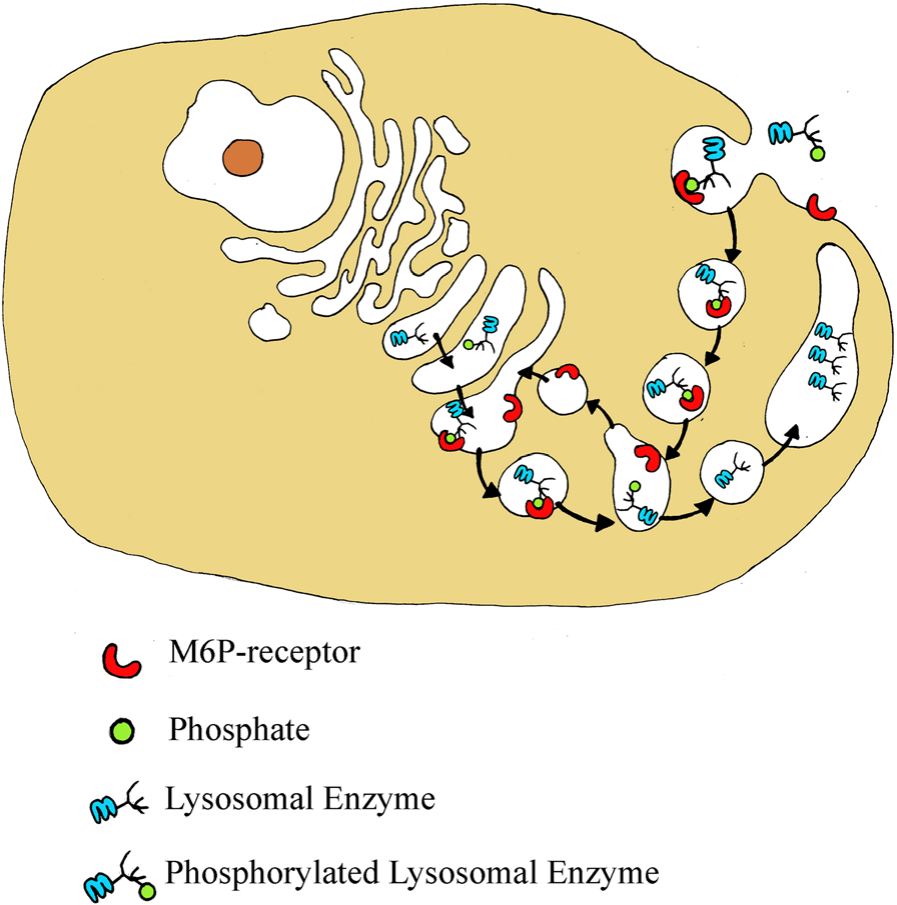
Mannose-6-phosphate (M6P) residues on the oligosaccharide chains of lysosomal enzymes are recognized by specific receptors present in the cell. Thanks to these receptors, the neo-synthesized enzymes are directed to the lysosomal compartment, where they perform their function. The M6P receptors are also expressed on the plasmatic membrane and this allows recombinant lysosomal enzymes to be “captured” by the cells and, following the pathway of the endocytic pathway, to be properly transported to the lysosome. Once lysosomes are reached, recombinant enzymes can replace the enzymatic deficit and degrade the accumulated substrate

The recombinant enzymes are produced in continuous human (fibroblasts) or animal cell lines (Chinese hamster ovary (CHO) cells) and plant cells [1] and are a purified form of the lysosomal enzymes. The resulting glycoproteins present mannose-6-phosphate (M6P) residues on the oligosaccharide chains. This allows specific binding of the enzyme to M6P receptors on the cell surface, thus enabling the enzymes to enter the cell and to be targeted to lysosomes, with subsequent catabolism of accumulated substrates [2] (Fig. 1).

The first effective treatment with ERT was performed in patients with Gaucher disease [3] and in the last 15 years ERT has become available for other lysosomal storage disorders including some types of mucopolysaccharidoses (MPS).

MPS I (Hurler, Hurler-Scheie, Scheie syndrome) was the first MPS type treated with ERT (available since 2003); subsequently the treatment became available for MPS VI (Maroteaux-Lamy syndrome; 2005), MPS II (Hunter syndrome; 2006), and MPS IVA (Morquio A syndrome; 2014) (Table 1). Recently, the recombinant enzyme β-glucuronidase has been tested for patients with MPS VII (Sly syndrome) [4, 5] and, to date, the treatment is available for commercial use in the United States where it was approved by the US Food and Drug Administration on 15 November 2017 (https://www.fda.gov/newsevents/newsroom/pressannouncements/ucm585308.htm accessed on 27 June 2018) and is under review by the European Medicines Agency (EMA) (EMA/CHMP/181307/2018 Committee for medicinal products for human use (CHMP) Draft agenda for the meeting on 23–26 April 2018).

**Table 1:** Enzyme replacement therapy (ERT) regimens for mucopolysaccharidoses (MPS)

	MPS I	MPS II	MPS IVA	MPS VI
Enzyme deficiency	α-l Iduronidase (IDUA)	Iduronate-2-sulphatase (IDS)	N-acetylgalactosamine-6-sulphatase (GALNS)	N-acetylgalactosamine-4-sulphatase (arylsulphatase B; ARSB)
Glycosaminoglycan accumulation	Dermatan sulphate and heparan sulphate	Dermatan sulphate and heparan sulphate	Keratan sulphate and chondroitin-6-sulphate	Dermatan sulphate
Drug	Laronidase (Aldurazyme®; Genzyme Europe B.V., Gooimeer 10, NL-1411 DD Naarden, The Netherlands), available since 2003	Recombinant human idursulphase (Elaprase®; Shire Human Genetic Therapies, Inc., Cambridge, MA, USA), available since 2006	Elosulphase alpha (Vimizim™; Bio Marin Pharmaceutical, Inc., Novato, CA, USA), available since 2014	Galsulphase (Naglazyme®; Bio Marin Pharmaceutical, Inc., Novato, CA, USA), available since 2005
Dosage	0.58 mg/kg body weight administered once every week as an intravenous infusion. The initial infusion rate of 10 μg/kg/h may be increased every 15 min, if tolerated, to a maximum of 200 μg/kg/h. The total volume of the administration should be delivered in approximately 3–4 h	0.5 mg/kg body weight administered once a week as intravenous infusions over 3 h. The duration of infusion can be shortened gradually to 1 h if there are no infusion-associated reactions (IARs)	2 mg/kg body weight administered once a week. The total volume of the infusion should be delivered over approximately 4 h	1 mg/kg body weight administered once a week as an intravenous infusion over 4 h
Official suggested premedication	With initial administration of Aldurazyme or upon re-administration following interruption of treatment due to previous IARs, pre-treatment with antihistamines and/or antipyretics approximately 60 min prior to the start of the infusion is recommended	Antihistamines and/or corticosteroids can be considered for those patients who have experienced previous IARs during the infusions	Patients should receive antihistamines with or without antipyretics 30 to 60 min prior to start of infusion	Antihistamines with or without antipyretics approximately 30–60 min prior to the start of infusion
Home treatment	Available	Available	Not available	Not available

Results from clinical trials and the real-world setting confirm the efficacy and safety of ERT in the treatment of these multisystem, progressive disorders [6]. The major proportion of the infused recombinant enzymes for MPS is delivered to the visceral organs such as the liver, kidney, and spleen [7, 8]. The infused enzymes have a short half-life in the circulation due to rapid binding to M6P receptors and uptake into visceral organs. It is known that only a small fraction of the recombinant enzyme can reach the bone cartilage and the eye, explaining why improvements of these organ/systems are limited even after long-term treatment [7, 9]. Moreover, due to the inefficacy of recombinant enzymes to cross the blood–brain barrier (BBB), there are no benefits of ERT for central nervous system (CNS) involvement [10, 11].

The ERT regimen for MPS requires weekly intravenous infusions of the recombinant enzyme. ERT is a life-long therapy, and each infusion takes 3 to 4 h depending on the enzyme and the dose (Table 1). There is the potential for severe infusion reactions; life-threatening anaphylaxis has rarely occurred in patients receiving ERT [12]. Most infusions are given in a hospital setting because of this risk, but home infusions are reported to be feasible and safe for some patients and thus home treatment is now available for selected patients with MPS I and MPS II [13, 14]. The feasibility of home therapy for any MPS patient should be based on a risk/benefit evaluation by the treating physician, the patient, and the patient’s caregiver.

A comprehensive search of journal articles regarding safety and effectiveness of ERT in MPS I, MPS II, MPS IV, and MPS VI from 2003 to July 2017 was carried out on PubMed. The subject headings were Mucopolysaccharidosis I, Mucopolysaccharidosis II, Mucopolysaccharidosis IV, and Mucopolysaccharidosis VI, MPS I, MPS II, MPS IV, MPS VI, enzyme replacement therapy, ERT, laronidase and Aldurazyme, idursulfase and Elaprase, elosulfase and Vimizim, galsulfase and Naglazyme. They were used alone and in combination. All the results of the clinical trials are reported and commented upon, while only the most relevant and/or interesting (in our judgement) clinical studies were considered in this review.

### Objectives of ERT

The various types of MPS have differences and similarities in their clinical pictures (see Galimberti et al. [15] and Rigoldi et al. [16] in this supplement) but we can generally say that the ideal aims of ERT are the same for all of them: reducing glycosaminoglycan (GAG) accumulation and organomegaly, improving growth (by ameliorating bone structure) and reducing bone deformities, improving the range of motion (ROM) of joints, and improving respiratory function, heart function, hearing, visual acuity, and quality of life (QoL). The major drawback of ERT molecules is their inability to cross the BBB and cure CNS pathology [10, 11].

### What are the major effects and limits of ERT in MPS?

#### GAG and organomegaly

The demonstration that ERT is biochemically effective is given by the impressive fast decline of urinary GAG concentration (uGAG) over the first 3–6 months of administration followed by a slow continuous decline during the following years [17–23]. From the clinical point of view, a prompt reduction in liver and spleen volumes is observed after few months of therapy, which is subsequently maintained [17–23]; this effect was somehow expected from the beginning considering that tissue distribution studies in animals [8, 24] had shown very high uptake of the recombinant enzyme in the liver and spleen. Reduction in liver size may be relevant for the outcome of patients because it can directly help in improving respiratory function through facilitating diaphragm excursions.

In summary, ERT is very effective in reducing urinary GAG to approximately normal values and improving liver and spleen size. This effect is sustained over time.

#### Joints

One of the major complaints of patients affected by MPS I, II, and VI is joint stiffness which hampers the easy execution of normal activities of daily life (combing, bathing, dressing, putting a hat on the head). MPS IVA patients instead have joint laxity and other different disturbances such as pectus carenatum, wrist subluxation, early presentation of genu valgum, and frequent osteoarthritis in adults [25]. Passive joint ROM improved in MPS I, II, and VI during clinical trials and improvement was maintained in the long term, although never reaching a normal extension/abduction of joints. Improvement is reported mainly for the shoulder, while the changes for the other joints have not been significant [12, 18, 19, 26–29]. The improvements in ROM were partial but allowed the accomplishment of many activities of daily living according to Sifuentes et al. [17] and Lampe et al. [19]. Although the majority of the authors agree that ERT has an effect, albeit limited, on joint stiffness, other papers report no effect of ERT on joint limitations [14, 30].

In summary, the effect of ERT on joint movement is probably variable from one individual to another, partial even after many years of ERT, is limited to the shoulder and does not significantly affect the other joints. Furthermore, the different responses to therapy may be explained by different joint conditions at the start of ERT [31].

#### Heart

Heart involvement is typical of MPS. GAG deposition in the myocardium and cardiac valves is the first step of a complex pathway starting with the release of pro-inflammatory cytokines and matrix metalloproteinases consequently activating the macrophages that ultimately damage the tissues [30]. While valve disease, when present at the start of ERT, is not reversible and progressively worsens, myocardial hypertrophy (or pseudohypertrophy) is responsive to ERT and the ejection fraction improves [20, 26, 27, 29, 31] (see also Boffi et al.in this Supplement [30]).

In summary, ERT improves the geometry and contraction of the cardiac muscle but has no clear effect on the valve structure.

#### Ear, nose, and throat, trachea, and pulmonary function

Ear, nose, and throat (ENT) disturbances are much frequent in MPS and consist of recurrent otitis and rhinosinusitis, tonsil and adenoid hypertrophy, sleep-related breathing disorders (oral breathing, snoring, obstructive sleep apnoea syndrome), and both conductive and sensorineural hearing loss [32–37]. Few data are reported about the effects of ERT on ENT signs and symptoms; ERT is acknowledged to reduce the number of upper airway infections and to improve sleep apnoea in the long term [12, 17, 19, 37, 38], mainly in patients with low-titre inhibitory antibodies [36]. Tomanin et al. [39], however, showed no effect of ERT on sleep apnoea in MPS II. Besides this, ERT does not seem to be very effective in reducing tonsil and adenoid hypertrophy or hearing deficit [20, 26, 40].

Spirometric tests evaluating forced expiratory volume in 1 s (FEV1) and forced vital capacity (FVC) (usually expressed as percent predicted FVC, or FVC%) have been used in clinical trials for all four MPS, showing improvements of 3–5% in the first year of treatment in MPS I and MPS IVA [23, 41]. For MPS II and MPS VI, FVC% did not significantly improve in double-blind trials [42–44]. In the long-term follow-up studies, results for FEV1 and FVC% range from stabilization to 11 ± 17% change from baseline [12, 18, 44, 45]. However, it seems that most patients probably reach a plateau after improvement at around 1–2 years of ERT and then stabilize or slowly progressively decline [12, 45]. The reason for this may be that ERT has effects only on one of the components responsible for airway insufficiency, which is multifactorial in MPS: GAG deposition in the soft tissues causes obstructive upper airway disease; tracheobronchial narrowing due to stenosis and malacia is responsible for obstructive lower airway disease; and chest deformities and poor mobility of the ribs lead to restrictive airway signs and symptoms. ERT is expected to be more effective on soft tissues and upper airway obstruction than on the other two factors. Chest deformities cannot be reverted, and the structure of the cartilaginous skeleton of the trachea and bronchi is likely to be marginally modified by the presently available ERTs [46]. Two recent papers address in detail the issue of tracheal and bronchial narrowing in MPS patients [47, 48]. In many of these individuals, a severe tracheal collapse during expiration is seen and, the longer they survive, the more frequent become the complications of bronchial and tracheal stenosis and malacia. These deformities are frequently the basis of the severe obstructive respiratory symptoms of adult MPS patients, mainly MPS I, II, and VI, and a satisfactory treatment still needs to be found [47, 48].

In summary, ERT partially improves the functional capacity of the lung with great variability in different individuals; probably the improvement is limited to the first years of treatment, reaching a plateau. ERT has no effect on the anatomic structure of the trachea and bronchi which are narrow and tend to collapse during expiration.

#### Endurance

Energy and endurance have been most commonly assessed in MPS-treated patients with the 6-min walk test (6MWT). Other tests of endurance are the 12MWT, used in the clinical trials for MPS VI, and the 3-min stair climb used in the trials for MPS VI and IV [23, 45, 49]. The 6MWT is a submaximal exercise tolerance test which includes evaluations of the responses and functional reserves of pulmonary, cardiovascular, and musculoskeletal systems [50]. An improvement in the 6MWT was seen after short-term treatment in clinical trials and in long-term studies for all four MPS, although in the very long term it seems they reach a plateau [12, 18, 31, 41, 42, 45, 49].

However, both spirometry and 6MWT can be performed only in patients who are not too young or cognitively impaired; there are therefore categories of patients for whom these parameters cannot be applied.

In summary, tests of endurance are much suitable for testing improvements attributable to ERT in clinical trials because their results are seen early, a few months after starting treatment. Their improvement is sustained over the subsequent years. However, only patients with no cognitive impairment and who are not too young are able to undergo these tests.

#### Bones and growth

Bio-distribution of ERT in bones, articular, and growth cartilage is modest, probably mainly due to their poor vascular supply [10, 12]. No effect on skeletal deformities was shown in clinical trials [23, 41, 42, 51]; it is generally agreed that bone disease cannot be reversed or even stabilized by ERT [52].

As for growth, there are reports showing improvement of growth after ERT in MPS I, MPS II, and MPS VI [53–55], but this effect is usually limited unless the patient is treated from the first weeks or months of life. This has been demonstrated in familial case reports of affected siblings where the earlier treated siblings had less skeletal deformities and better growth than the first sibling [19, 56–62].

These family cases show that ERT may have an effect on growth and bone development if started very early. An improvement in growth during ERT has also been demonstrated in Morquio A patients under 5 years of age who were included in the open-label ERT MOR-007 trial [63].

In summary, the effect of ERT on bones and cartilage is limited, probably partially due to scarce penetration. However, a very early ERT start seems to improve bone health and growth as demonstrated by studies on siblings [64].

#### Eyes

Eyes are frequently involved in the clinical picture of MPS. Corneal clouding is more often reported in MPS I, VI, and IVA, and optic disk swelling, optic atrophy, papilloedema, and retinal pigment degeneration in all [65]. Few data are available on the efficacy of ERT. Stabilization and improvement of photophobia and, in some cases, improvement of visual acuity and reversal of papilloedema have been reported. It seems that if there are improvements, they are partial and possibly variable among individuals [33, 66–70].

In summary, some patients had an improvement in photophobia and visual acuity and other eye problems after ERT, but this is not observed in most patients.

#### Health-related quality of life (HRQoL)

The demonstration of biochemical and clinical improvements after ERT with all the assessments described above does not clarify if these effects really mean an improvement in QoL for patients and their families. Is it relevant for patients having a mild improvement in pulmonary function tests or in metres walked at the 6MWT? For the patients it is probably more relevant and meaningful to be more autonomous in performing activities of daily living (ADL), having less pain and satisfactory relationships with schoolmates or in the working environment. With the purpose of exploring this area, many studies have included the assessment of ADL, HRQoL, and pain in the parameters evaluated to demonstrate the efficacy of ERT. A recent review reports a critical comment on all the published studies and the different tests used [44]. The most frequently used test was MPS-HAQ (CHAQ), an adaptation of the Health Assessment Questionnaire (HAQ)/Childhood Health Assessment Questionnaire (CHAQ) test used for rheumatoid arthritis [71]. ADL and HRQoL were reported to improve after long-term ERT in MPS I patients who underwent clinical trials [12, 17]. The MPS-HAQ disability index improved after long-term ERT in cognitively normal MPS II patients [18, 20]. MPS-HAQ/CHAQ and pain control also improved in MPS IVA and MPS VI patients [21, 23, 26, 29, 51, 72–75]. However, the vast majority of the patients in these studies did not have cognitive delay. Demonstration of improvement in these parameters in the patients with CNS involvement (i.e. the most severe forms of MPS I and II) is lacking [20].

In summary, ERT is effective in improving ADL, HRQoL, and pain in those patients with no cognitive delay. We do not have enough data on the more severe patients with MPS I and II.

#### CNS

It is generally accepted that all the intravenous ERTs developed for MPS and other lysosomal storage diseases do not reach the CNS in amounts sufficient to prevent deterioration of CNS and neurocognitive functions [7, 11, 76]. This is particularly true for MPS I and MPS II, the MPS types with CNS involvement in the majority of the patients. The role of ERT in MPS I and MPS II severe phenotypes has been subject of debate [38, 77]. For MPS I, the main point is “when you should not offer HSCT to an MPS I Hurler patient balancing risks of HSCT with forecasted results?”

On the basis of the progressively reduced harm and mortality of HSCT in recent years, it is at present performed even beyond 2.5 years of age and in MPS I Hurler-Scheie patients who have a slower decline of cognitive functions [77–80]. For MPS II, the option of HSCT is not recommended at present, although a recent paper shows better results compared to ERT in a considerable number of patients [81]. Most MPS II severe patients thus receive ERT from the diagnosis. The treatment is usually decided together with the family taking into consideration advantages for somatic organs and disadvantages related to possible infusion reactions and worsening of behavioural disturbances due to veni-puncture every week followed by 4-h infusion treatment. In our personal experience, only 2 of 19 families refused starting ERT in their children with a severe form of MPS II. This is consistent with the results of a survey conducted in MPS families where 77% of respondents were in favour of starting ERT in a patient with a severe phenotype, even knowing that treatment cannot alter the intellectual deterioration associated with the disease [82]. At present, the opinion of the experts is that “withholding a therapeutic that has the potential to improve some of the somatic manifestations of the disease because of an eventual cognitive decline”, or even “if the cognitive decline is manifest”, “is not justifiable” [38]. The response to ERT would be periodically assessed after starting treatment and, in case of apparent lack of clinical benefit, the decision to withdraw will then be discussed with the family [38].

In summary, ERTs developed for MPS and other lysosomal storage diseases do not reach the CNS in amounts sufficient to prevent deterioration of the CNS and neurocognitive function.

### Safety and immunogenicity

#### Safety

Based on clinical trials, ERT for MPS is considered well tolerated and has an acceptable safety profile. Infusion adverse reactions (IAR), such as rash, urticaria, angioedema, bronchoconstriction, rhinitis, and anaphylaxis, have been reported in approximately 50% of MPS I patients treated with laronidase [12], approximately 30% of MPS II patients treated with idursulphase [83], approximately 90% of MPS IVA patients treated with elosulphase [23], and approximately 50% of MPS VI patients treated with galsulphase [84]. The majority of IARs are usually mild and/or successfully treated by interrupting or slowing the rate of infusion and/or by the administration of anti-histamines, antipyretics and/or corticosteroids. Most patients who experience an IAR receive and tolerate subsequent infusions. Serious adverse reactions have been rarely reported such as anaphylaxis requiring emergency tracheotomy for an associated airway obstruction in a 16-year-old patient with MPS I Hurler-Scheie after 44 laronidase infusions [12]. The reactions experienced during ERT can be caused by either IgE-mediated or non-immunological mechanisms. In the case of recurrent IARs, with failure of pre-medication to prevent hypersensitivity reactions, desensitization is indicated. Effective desensitization has been reported in patients affected by MPS I, MPS II, and MPS VI [85, 86].

#### Immunogenicity

Most ERTs used for treating lysosomal storage disorders produce an anti-drug antibody (ADA) response which can potentially reduce efficacy or lead to hypersensitivity reactions. The enzymes are taken up by antigen-presenting cells which process and present them to helper T cells specific for the generated peptide. Helper T-cell signals activate antigen-specific B cells to proliferate and differentiate into memory B cells, and into antibodies secreting plasma cells. ADAs may impair the desired biological effects of the therapeutic enzyme through several mechanisms, including altered enzyme targeting, increased enzyme turnover, and/or inhibition of the catalytic site. They can bind to segments of the therapeutic enzyme that are not associated with particular functional activities (non-neutralizing antibodies) or bind to the uptake or catalytic domains (neutralizing antibodies). The level and nature of the residual endogenous enzyme affects the propensity of the patient to generate ADAs. More than 90% of MPS I patients developed antibodies to laronidase during the first few months of treatment [12], about 50% of MPS II patients produced antibodies against idursulphase [22], and almost all patients treated with elosulphase [87] and galsulphase [88] produced ADAs. A clear correlation between ADA titre and clinical outcome has been shown in infantile-onset Pompe disease [89]; less is known about the role of immunogenicity in MPS, and the possible interference of antibodies with the efficacy of ERT is still unclear. A relationship between exposure to ADAs and a pharmacodynamic biomarker, uGAG, has been demonstrated in MPS I and MPS II. Some authors analysed the role of inhibitory antibodies on metabolic biomarkers and sleep disorders in ERT-treated MPS I patients. They showed that increasing inhibition of enzyme activity by antibodies correlated significantly with poorer substrate reduction [36].

A case of allo-immune membranous nephropathy has been reported in a patient with MPS VI treated with galsulphase. The finding of high titres of circulating ADA, which peaked at the onset of the nephrotic syndrome, indicates a mechanism of allo-immunization against the recombinant enzyme [90].

Undoubtedly, the effect of ADAs in MPS is more difficult to evaluate than in infantile Pompe disease due to the slowly progressive course of MPS and the fact that no consistent relationship between ADA titre and clinical outcome has been documented until now. A possible role of the time of uptake of the drug from the plasma into target cells via the M6P receptor, and therefore the mean plasma half-life of distinct ERT, has been recently hypothesized [86]. Laronidase has a mean plasma half-life ranging from 1.5 to 3.6 h, while idursulphase, galsulphase, and elosulphase exhibit a mean plasma half-life of 44, 26, and 36 min, respectively. This rapid uptake may limit the drug’s exposure to antibodies in the plasma and might reduce the formation of immune complexes and their downstream effects.

Some attempts at immune tolerance induction in MPS patients treated with ERT have been performed. An open-label, phase II trial was undertaken to determine the safety and effectiveness of a prophylactic immunosuppressive regimen (cyclosporine and azathioprine) in treatment-naive patients with severe MPS I caused by two nonsense mutations [91]. Unfortunately, the study was terminated early due to changing standards of care for this patient population with inconclusive results. An immune tolerance induction regimen similar to that used in infantile Pompe disease patients has been used in a 4-year-old MPS II patient with sustained high antibody titre and limited clinical efficacy of idursulphase treatment. Over 18 months, therapy with atumumab, bortezomib, methotrexate, short-term dexamethasone, and IVIG resulted in a significant reduction in neutralizing anti-idursulphase IgG titre and a moderate reduction in uGAG levels compared with baseline, while modest clinical improvements were observed [92].

Real-time access to ADA testing is not always easy in the clinical setting and the time to obtain assay results may reduce its clinical utility. However, the reader is reminded that ERT is a lifelong therapy for a devastating disease and that routine monitoring of ADAs is essential and should be part of the routine management of each patient on ERT.

Further prospective and more detailed investigations are needed to understand the real impact of the immune response to ERT and therefore the long-term safety and efficacy. Furthermore, more studies are needed to evaluate the type and the risk/benefit ratio of immunosuppressive therapy.

In summary, ERT for MPS is considered well tolerated and has an acceptable safety profile. However, the real impact of immune response on long-term efficacy remains to be elucidated.

## Conclusions

Experience with ERT in MPS I, II, and VI is now reaching more than 10 years, while in MPS IVA the time of observation is shorter. As clearly reported in the literature, we could observe the benefits of this treatment in our patients in terms of reduction of organomegaly, improving pulmonary and heart function, and ameliorating HRQoL, but we also progressively realized that the patients had a big burden of “residual disease” accompanied by the need for several surgical treatments with increased risk of anaesthesia and bone and cartilage abnormalities which are not cured. This implies a risk of cord compression at any time and progressive tracheo-bronchomalacia with severe obstructive lower airway disease. Many patients develop antibodies to the recombinant enzyme and it is not clear yet if high-titre antibodies might influence the efficacy of the treatment with ERT [36].

The use of ERT in cognitively involved patients is a subject of debate and the most accepted position in the scientific community at present is that ERT should be started in any patient because it has the potential to improve some of the somatic manifestations of the disease [38].

Over the years we have also learned from many anecdotal familiar reports that ERT is much more effective, even on the bones, if administered very early. Since ERT is more powerful if it is started early, in a pre-symptomatic phase, new-born screening of treatable MPS has been proposed and pilot studies have been developed in many countries [93]. Whether starting ERT at a very precocious age would be able to halt the progression of all the signs of the disease (excluding as usual the CNS) is not known. For certain, in the case of treatment of a pre-symptomatic or oligo-symptomatic patient, other ethical questions would arise, such as how to distinguish between severe and attenuated forms which might deserve different treatments (HSCT vs ERT for example). The elevated costs of these treatments complicate the choice.

Other drugs are at present being developed: different kinds of more powerful ERT, specifically targeting tissues such as bones where the disease is prominent, drugs based on different principles to enzyme replacement such as substrate deprivation, chaperone therapy, exon skipping, and gene therapy [94, 95]. We hope that all these research lines will develop good treatments for MPS to be used alone or associated with ERT.

## Abbreviations

ADA: Anti-drug antibody
ADL: Activities of daily living
BBB: Blood–brain barrier
CHAQ: Childhood Health Assessment Questionnaire
CHO: Chinese hamster ovary
CNS: Central nervous system
ENT: Ear, nose, and throat
ERT: Enzyme replacement therapy
FDA: Food and Drug Administration
FEV1: Forced expiratory volume in 1 s
FVC: Forced vital capacity
GAG: Glycosaminoglycan
HAQ: Health Assessment Questionnaire
HRQoL: Health-related quality of life
HSCT: Haematopoietic stem cell transplantation
IAR: Infusion adverse reactions
M6P: Mannose-6-phosphate
MPS: Mucopolysaccharidosi(e)s
QoL: Quality of life
ROM: Range of motion
uGAG: Urinary glycosaminoglycan
